# Uptake of Aggregating Transthyretin by Fat Body in a *Drosophila* Model for TTR-Associated Amyloidosis

**DOI:** 10.1371/journal.pone.0014343

**Published:** 2010-12-16

**Authors:** Malgorzata Pokrzywa, Ingrid Dacklin, Monika Vestling, Dan Hultmark, Erik Lundgren, Rafael Cantera

**Affiliations:** 1 Department of Molecular Biology, Umeå University, Umeå, Sweden; 2 Department of Clinical and Experimental Medicine, Linkoping University, Linkoping, Sweden; 3 Institute of Medical Technology, University of Tampere, Tampere, Finland; 4 Department of Zoology, Stockholm University, Stockholm, Sweden; 5 Developmental Neurobiology, Instituto de Investigaciones Biológicas Clemente Estable (IIBCE), Montevideo, Uruguay; Ohio State University, United States of America

## Abstract

**Background:**

A functional link has been established between the severe neurodegenerative disorder Familial amyloidotic polyneuropathy and the enhanced propensity of the plasma protein transthyretin (TTR) to form aggregates in patients with single point mutations in the TTR gene. Previous work has led to the establishment of an experimental model based on transgenic expression of normal or mutant forms of human TTR in *Drosophila* flies. Remarkably, the severity of the phenotype was greater in flies that expressed a single copy than with two copies of the mutated gene.

**Methodology/Principal Findings:**

In this study, we analyze the distribution of normal and mutant TTR in transgenic flies, and the ultrastructure of TTR-positive tissues to clarify if aggregates and/or amyloid filaments are formed. We report the formation of intracellular aggregates of 20 nm spherules and amyloid filaments in thoracic adipose tissue and in brain glia, two tissues that do not express the transgene. The formation of aggregates of nanospherules increased with age and was more considerable in flies with two copies of mutated TTR. Treatment of human neuronal cells with protein extracts prepared from TTR flies of different age showed that the extracts from older flies were less toxic than those from younger flies.

**Conclusions/Significance:**

These findings suggest that the uptake of TTR from the circulation and its subsequent segregation into cytoplasmic quasi-crystalline arrays of nanospherules is part of a mechanism that neutralizes the toxic effect of TTR.

## Introduction

The transthyretin (TTR) amyloidoses represent a group of human diseases in which misfolded molecules of TTR aggregate and cause damage to surrounding tissues. Depositions of TTR-derived amyloid are found widespread in heart, kidney, eye and other organs [1], [2] indicating the systemic and heterogeneous nature of these disorders. Most symptoms in patients, however, arise from peripheral and autonomic nerve dysfunction associated with formation of TTR fibrils along the nerves. Unlike Alzheimer's disease, another common amyloidosis, localized TTR-derived amyloid in the central nervous system is rarely reported [3], [4].

TTR is one of the 27 human proteins known to be associated with local or systemic amyloidosis [5]. TTR is a soluble protein primarily synthesized in the liver, the choroid plexus and the retina and secreted to plasma, cerebrospinal fluid and vitreous humour, respectively. The native molecule, assembled as a 55-kDa homotetramer, functions as a transport protein of the thyroid hormone thyroxine and retinol (vitamin A), the latter through association with retinol-binding protein [6], [7]. TTR has, however, an inherent propensity to assemble into insoluble amyloid fibrils, which sporadically leads to senile systemic amyloidosis (SSA) with deposits of wild-type TTR (TTRwt) mainly in the cardiac tissue late in life [8]. Familial forms of TTR-related amyloidosis are inherited in an autosomal dominant manner and arise from single point mutations in the coding sequence of the gene. Today, there are over 100 variants of TTR described, of which the majority is amyloidogenic [1]; (for summary see http://www.bumc.bu.edu/Dept/Content.aspx?DepartmentID=354&PageID=8850). Depending on the primary site of deposition, the disease has been termed familial amyloid cardiomyopathy (FAC) or familial amyloid polyneuropathy (FAP).

Amyloidogenic mutations in the TTR gene are known to destabilize the quaternary structure of the molecule, which leads to tetramer dissociation and partial subunit unfolding. This process results in the accumulation of misfolded TTR monomers that self-assemble into oligomers and then amyloid fibrils [9], [10], [11].

Similarly to other amyloids, mature TTR amyloid fibrils are highly organized, insoluble structures that become resistant to proteolysis. In the electron microscope they show a well-defined ultrastructure of rigid and non-branching fibres, approximately 11–13 nm in diameter and consist of 4 protofilaments [12]. In the last couple of years, the focus of research into amyloid toxicity has shifted towards the small oligomeric aggregates formed on or off the fibrillogenesis pathway, since these structures have been suggested to be the main mediators of pathogenicity [13]. Toxic species of TTR made of recombinant protein have been identified *in vitro*; as well as *in vivo* in TTR transgenic mice and in *ex vivo* explants from FAP patients [14], [15], [16], [17]. While much is known about the structure of different amyloids, there is no evidence on correlation between the final deposits and severity of the disease [18], [19]. Similarly, *in vitro* studies on TTR aggregation has brought a huge insight into understanding of amyloid formation, but the exact process of TTR amyloid fibrils formation *in vivo* is not known [5].

In a previous work, we have generated a transgenic model for TTR-associated amyloidosis in the fly *Drosophila melanogaster* by overexpressing either normal TTR (TTRwt), the clinically relevant mutant TTRL55P, or the highly destabilized engineered mutant TTR-A (TTRV14N/V16E; [20]), all in secreted form and thus detectable in hemolymph [21]. The expression of mutated TTR, but not wild-type TTR, caused a complex phenotype partially reminiscent of the human pathology, which included shortened life span, neurodegeneration and early locomotor dysfunction. An interesting feature was an abnormal posture of the wings termed “dragged-wing phenotype”. Surprisingly, this phenotype had higher penetrance (40 to 99%) in flies with a single copy of the transgene than in flies with two copies (less than 40%).

To resolve this apparent paradox we decided to explore the TTR expression pattern in detail by both an ultrastructural analysis of the affected tissues and functional analysis of aggregates. The results firmly establish a correlation between the expression of the transgene coding for the mutated TTR-A and the generation of intracellular aggregates of nanospherules and nanofilaments in brain glia and thoracic adipose tissue, with more and larger aggregates in old flies carrying two copies of mutated TTR. Moreover, an *in vitro* assay with the human neuroblastoma cell line IMR-32 showed less toxicity when the material originated from older flies, suggesting that the intracellular formation of aggregates is part of a mechanism for the neutralization of the toxic effects of TTR-A. This opens a new avenue for the experimental elucidation of the relationship between toxicity and the different forms of soluble or aggregated TTR proteins, validating the potential of this *Drosophila* model.

## Results

### Localization of TTR aggregates and amyloid filaments in transgenic flies

All the transgenic flies used in this study were constructed with a *GMR*-*Gal4* driver to ensure strong and specific expression in the retina throughout postembryonic stages [21]. This gal4 driver is also expressed to some degree in a few other tissues, but not in the fat body (Figure S1). Since the TTR protein encoded by the transgene contains a signal peptide, secretion from the retina will deliver the protein directly to the circulatory system. Insects have an open circulatory system, with the brain, muscles, nerves and other tissues being surrounded by the blood, called hemolymph. Thus, besides exerting an effect in TTR expressing organs [21], other tissues could be affected. Several aspects of the phenotype, in particular the abnormal wing posture and flight defects [21], persuaded us to investigate the thoracic flight muscles with corresponding nerves and surrounding tissues. For practical reasons we focused on the dorsal thorax, preparing samples that contained some of the major flight muscles with attached motor neurons, sensory axons, fat body (adipose tissue), tracheae (respiratory tubes), hemocytes (blood cells) and epidermis.

**Figure 1:**
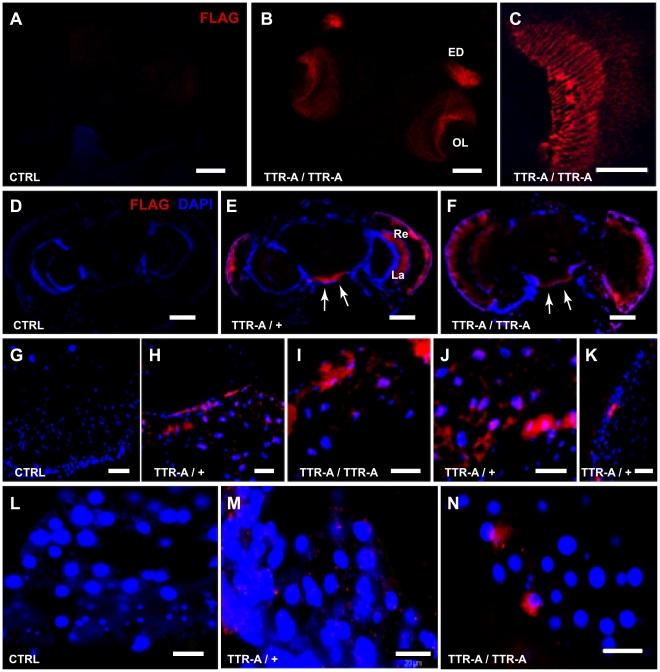
TTR localization pattern in transgenic flies. Immunodetection of FLAG-TTR (red in A–N) with nuclear counterstaining (blue in D–N) on cryo-sections is shown. Third instar larval CNS with eye discs of control (**A**), and ^FLAG^TTR-A/^FLAG^TTR-A (**B**, **C**) larvae show that the staining was specific for FLAG-TTR–expressing animals and restricted to the eye disk (ED) and Optic Lobe (OL) as expected for the *GMR-Gal4* driver. A detail of FLAG-TTR expression in the terminals of retinal photoreceptors inside the OL of the brain is shown in C. Horizontal head sections of 14 days old control (**D**), ^FLAG^TTR-A/+ (**E**) and ^FLAG^TTR-A/^FLAG^TTR-A (**F**) adult flies showing FLAG-TTR localization in the retina (Re) and lamina (La) as well as in perineurial glia (arrows). The staining was absent in the head fat body of control flies (**G**) and present in ^FLAG^TTR-A/+ (**H, J**), and ^FLAG^TTR-A/^FLAG^TTR-A (**I**) flies. Detail of retina with TTR-A aggregates in ^FLAG^TTR-A/+ flies (**K**). Thoracic fat body of control (**L**), and ^FLAG^TTR-A/+ (**M**), and ^FLAG^TTR-A/^FLAG^TTR-A flies (**N**). Aggregates of TTR-A were found in the retina and in fat body of head and thorax (Red “spots” in H–J and M–N). Scale bars, 100 µm in A–B, and D–F; 50 µm in C; and 20 µm in G–N. For a complete definition of the genotypes see Material and Methods.

To follow the expression pattern of TTR protein and its subsequent localization after secretion, we generated transgenic flies in which TTR was N-terminally tagged with a synthetic 8-amino-acid long FLAG epitope (DYKDDDDK; for details see M&M). We tested that the FLAG-TTR had the expected molecular size shift in control experiments. (Figure S2). Immunostaining with an antibody specific for the FLAG epitope gave no staining in control larvae (Fig. 1A) and a strong immunostaining in the optic lobes and eye disk of full-grown ^FLAG^TTR-A larvae (Fig. 1B), with staining in the photoreceptors as expected (Fig. 1C). In cryo-sections of 14 days old fly heads, the anti-FLAG staining (Fig. 1E, F) reproduced previous reports with anti-TTR antibodies [21], with no staining in control flies (Fig. 1D) and immunofluorescence in the retina and lamina (a visual brain centre postsynaptic to retinal axons) of adult ^FLAG^TTR-A/+ (Fig. 1E, K) and ^FLAG^TTR-A/^FLAG^TTR-A flies (Fig. 1F). We also observed FLAG-specific fluorescence in a rim around the subesophageal ganglion (arrows in Fig. 1E and F) where nuclei of perineurial glia are found [22].

Interestingly we found the FLAG positive signal in the head fat body surrounding the brain of ^FLAG^TTR-A/+ (Fig. 1H, J) and ^FLAG^TTR-A/^FLAG^TTR-A flies (Fig. 1 I) but not in control flies (Fig. 1G). Moreover, a distinct punctuated pattern of FLAG fluorescence was present in the thoracic fat body of ^FLAG^TTR-A/+ samples (Fig. 1M) and less frequent but larger in ^FLAG^TTR-A/^FLAG^TTR-A samples (Fig. 1N). Confocal microscopy revealed that the immunopositive puncta ranged in size between 0,3–0,99 microns in ^FLAG^TTR-A/+ samples and 0,79–9,49 microns in ^FLAG^TTR-A/^FLAG^TTR-A samples (A representative TTR-A aggregate in ^FLAG^TTR-A/^FLAG^TTR-A is shown in Figure S3). Such staining was neither detected in control samples (Fig. 1L) nor in wild-type Oregon flies (data not shown). Analogous findings on TTR localization were obtained in samples immunostained with anti-TTR antibodies, with a positive signal in adipose tissue of head and thorax, but neither muscles, nerves or other tissues (Figure S4).

**Figure 2:**
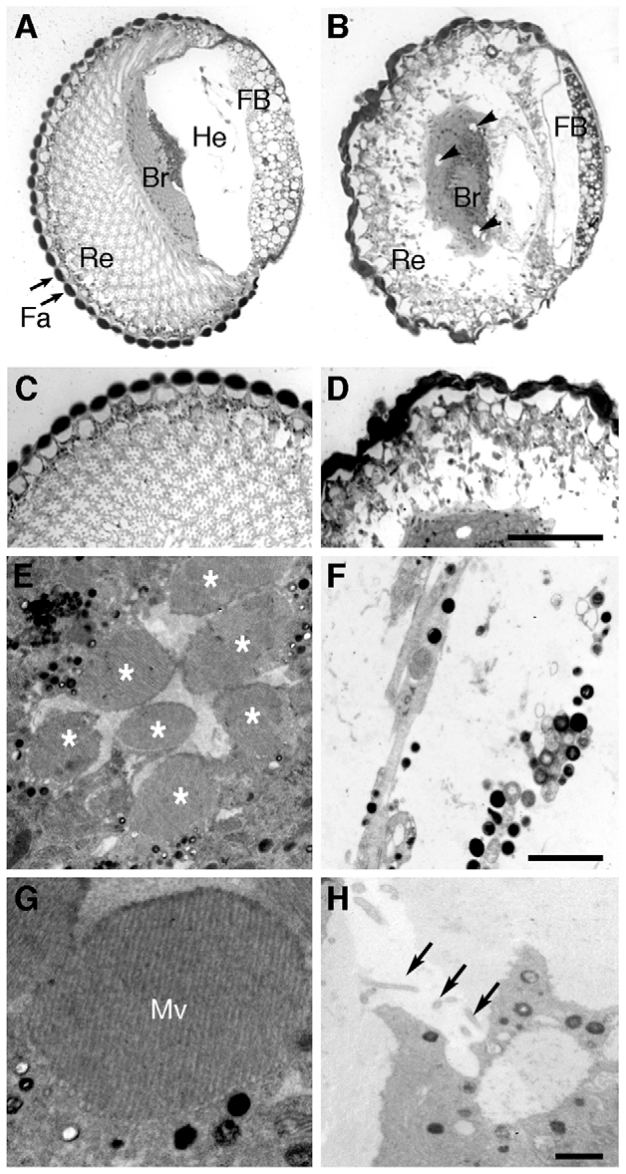
Histology (A–D) and ultrastructure (E–H) of the head in 14 days old flies. In sagital sections the facets (Fa), retina (Re), brain (Br) and fat body (FB) with a large space that in the living fly is filled with hemolymph (He) were clearly seen in wild-type (**A**) and TTRwt/TTRwt flies (not shown here, see Figure S5). TTR-A/TTR-A (**B**) and TTR-A/+ flies (Figure S5) flies had advanced retinal degeneration and brain vacuolation (arrowheads). TTR-A/TTR-A flies had also thinner fat body than flies of all other genotypes. Larger magnifications of the retina in wild type (**C**) and TTR-A/TTR-A flies (**D**) illustrate the massive degeneration of the retina in TTR-A/TTR-A flies (scale bar = 50 µm in C and D). With electron microscopy the normal ultrastructure of ommatidia was found in TTRwt/TTRwt flies (**E**) compared with wild-type flies (not shown), with a central core of seven retinal photoreceptors (asterisks in E) surrounded by support cells. In TTR-A/TTR-A flies the ommatidia were largely disrupted and many cells were missing, leaving instead empty spaces (**F**). Scale bar = 2 µm in E and F. At higher magnification, the tightly packed microvilli (Mv) characteristic of wild-type photoreceptors was found in TTRwt/TTRwt flies (**G**) but only very few microvilli were found in TTR-A/TTR-A flies (**H**, arrows). The scale bar in H shows 500 nm in G and H.

Light microscopy of toluidine-blue stained sections of fly heads was used to compare the histology of retina and brain in young (3 days old) and old (14 days old) flies. Retinal degeneration and sparse signs of brain vacuolation were detected in TTR-A/+ and TTR-A/TTR-A flies (Fig. 2B, D and Figure S5) as previously reported [21], but not in control flies (Fig. 2A, C and Figure S5).

In the thorax, there was no obvious histological pathology in muscles or other tissues. The exception was the fat body, which appeared hypotrophied and contained, only in old TTR-A/TTR-A flies, intracellular, acidophilic inclusions of rounded or ovoid shape and up to several microns in size.

**Figure 3:**
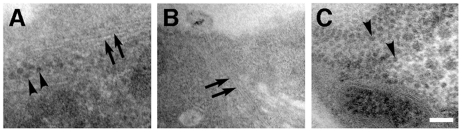
TEM analysis of brain glia in TTR-expressing flies. Arrays of nanospherules (arrowheads) and nanofilaments (arrows) were detected in the cytoplasm of glial cells of 14 days old TTRwt/TTRwt (**A**) and TTR-A/ TTR-A (**B**, **C**) flies. Scale bar = 100 nm.

When these samples were analyzed with transmission electron microscopy, the absence of obvious degenerative ultrastructural features was confirmed in muscle and nerves, in all genotypes and age groups (Figure S6). The fly retina is composed of distinct optic units called ommatidia, each formed by a central cluster of photoreceptors surrounded by support cells [23] (Fig. 2E). Empty spaces, corresponding in shape and size to single ommatidia, were detected in the retina of TTR-A/TTR-A flies of the young group (not shown). In the old TTR-A/TTR-A flies, this had advanced markedly and most ommatidia appeared disrupted or missing (Fig. 2F). The apical membrane of the insect retinal photoreceptors forms an array of tightly and regularly packed microvilli (asterisks in Fig. 2E, detail in Fig. 2G). At the stage of massive retinal degeneration the few remaining photoreceptors (compare Fig. 2E with F) had none or only few and short microvilli (Fig. 2H). On the other hand, the size and organization of the retina in flies expressing TTRwt (Fig. 2C, E) appeared identical to that of normal control flies independent of age. Arrays of nanofilaments 7–9 nm in thickness and nanospheres of about 20 nm in diameter (from here on termed “spherules”), were abundant in the cytoplasm of glial cells that cover the surface of the retina (subretinal glia, [22]) and those that cover the surface of the brain (perineurial glia, [22]) in TTRwt/TTRwt (Fig. 3A), TTR-A/+ and TTR-A/TTR-A flies (Fig. 3B, C), but not in wild type flies. These two types of glia are easy to recognize by EM because they are more electron-dense than neurons, they form the outermost layer of the brain and they are covered by an extracellular collagen matrix [22]. None of these structures were detected in brain neurons, although sporadic vacuolation (Fig. 2B), accumulation of phagosomes and lamellar bodies, all indicative of neurodegeneration, were scored in the brain of the old TTR-A/TTR-A flies (data not shown).

**Figure 4:**
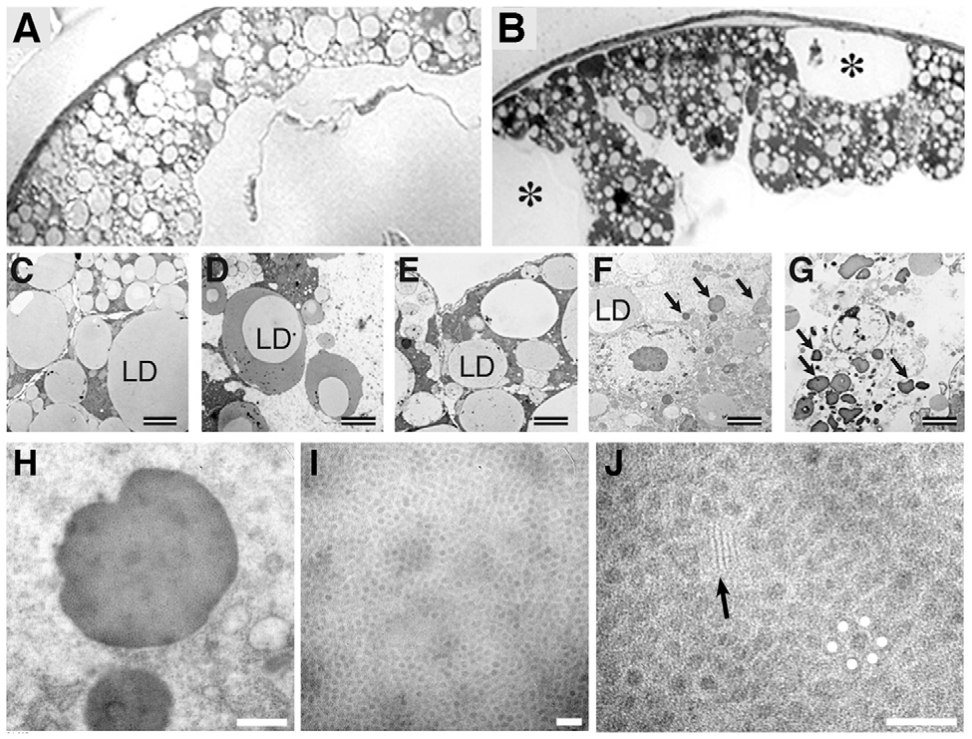
TEM analysis of the fat body in TTR-expressing flies. At the histological level the thoracic fat body of TTRwt/TTRwt flies had normal appearance compared with that of wild-type flies at both 3 (not shown) and 14 days of age (**A**) but in 14 days old TTR-A/TTR-A flies (**B**) it appeared to contain “holes” with the size and shape of single fat body cells (asterisks). Proper ultrastructure of the fat body was detected in wild-type (**C**), TTRwt/TTRwt (**D**) and TTR-A/+ (**E**) flies with small and large lipid droplets (LD) and normal cytoplasmic organization. In TTR-A/TTR-A flies (**F** and **G**), on the contrary, this tissue contained fewer lipid droplets and large numbers of dark bodies (arrows). The scale bar shows 2 µm in C–G. Some of the fat body cells containing more and larger dark bodies appeared to be disrupted (**G**, “bursting cell”). Measurements of the dark bodies at higher magnification showed that some of them were several microns in diameter (**H**) and were full-packed with spherules, about 20 nm in diameter and arranged with hexagonal pattern (**I**). In J, a white dot was marked digitally at the centre of each of the six spherules equidistant to a central, non-marked spherule to highlight this pattern. Small arrays of short, unbranched nanofilaments were occasionally found among the spherules (arrow in J). All the images shown here are from 14 days old flies and the scale bars represent 500 nm in H and 100 nm in I and J.

The most striking alteration besides the retinal degeneration was hypotrophy of the fat body in old TTR-A/TTR-A flies (compare Fig. 2A with B and Fig. 4A with B), reflected by decreased size, fewer lipid droplets and empty spaces which by shape and size suggested missing fat body cells (Fig. 4B, asterisks). In contrast, young TTR-A/TTR-A flies, as well as TTR-A/+ and TTRwt/TTRwt flies of any age had normal ultrastructure as seen in the wild type controls (Fig. 4 C–E). In the thoracic fat body of old TTR-A/TTR-A flies we found large cytoplasmic aggregates (arrows in Fig. 4 F and G) that were enclosed by a membrane, similar to other protein granules usually stored in this tissue. They reached sizes in a range of a few microns up to 6.2 µm. These aggregates were exclusively restricted to the fat body cells of old TTR-A/TTR-A flies and correlated with enlarged rough endoplasmic reticulum cisternae and frequently with apoptotic nuclei. Some of the fat body cells with largest accumulations of aggregates exhibited damaged cell membrane as if they were disintegrating (Fig. 4G).

### Ultrastructure of the aggregates formed in thoracic fat body of TTR-A/TTR-A flies

At very high magnification (200.000×), the aggregates consisted of membrane-bound bodies with a light matrix full-packed with spherules of about 20 nm in diameter (Fig. 4H–J). These spherules were arranged in an almost perfect hexagonal pattern, each surrounded by six equidistant spherules (Fig. 4J). At 300.000× TEM magnification, single spherules appeared to consist of a cortex of electron-dense “dots” of approximately 2–3 nm in diameter surrounding a less electron-dense core (data not shown). Inside some of the biggest aggregates we found unbranched filaments of 7–9 nm in thickness (arrow in Fig. 4J).

### Soluble aggregates of TTR-A are toxic to neuronal cells

**Figure 5:**
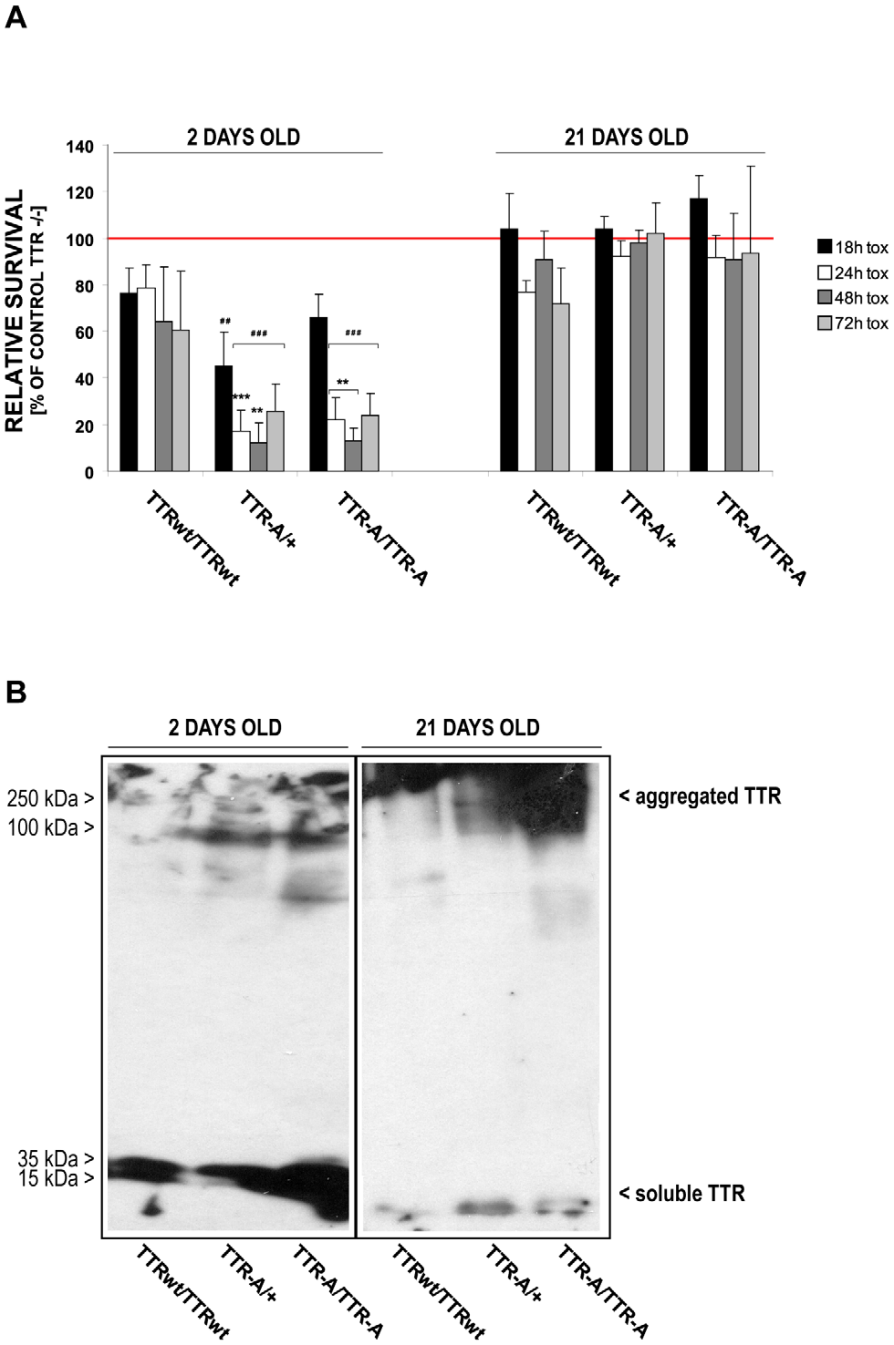
Neurotoxic effect of TTR-A to human cells in a time-course experiment. (**A**) Significant reduction of cell viability was detected in IMR-32 neuroblastoma cells exposed to TTR-A-containing extracts prepared from young vs. old flies (***P<0.001 for most of the time points; one-way ANOVA, sequential Bonferroni post-hoc). Cell viability was significantly reduced already 18h after exposure to the TTR-A/+ extracts (*^# #^ P* = 0.005, TTR-A/+ vs. control TTR−/−) and decreased over time in cells exposed to TTR-A/+ and TTR-A/TTR-A but not TTRwt/TTRwt extracts (*^# # #^ P*<0.001, TTR-A/+ and TTR-A/TTR-A vs. control TTR−/− for 24–72h). The extracts prepared from TTR-A/+ and TTR-A/TTR-A flies showed significant neurotoxic properties compared to the extracts from TTRwt/TTRwt flies (*** *P*<0.001, after 24h, ** *P*<0.01 after 48h for TTR-A/+ vs. TTRwt/TTRwt extracts; ** *P*<0.01 after 24–48h for TTR-A/TTR-A vs. TTRwt/TTRwt extracts). Red line indicates normalized viability of control cells exposed to TTR−/− extracts. (**B**) Age-dependent aggregation of TTR detected in fly extracts. TTR detected in extracts from young flies is mostly soluble (left panel), whereas in 21 days old flies aggregates are formed, which hardly migrate in the gel (right panel). TTR immunodetection was performed with TTR specific polyclonal antibody. Soluble TTR is represented by monomers and tetramers (double band), aggregated TTR consist of assemblies above 100 kDa.

We also investigated whether fly extracts enriched in hemolymph and fat body content (see M&M) affected cell viability of the human neuroblastoma IMR-32 cells (Fig. 5A). In a time-course experiment, extracts from 2 days old TTR-A/TTR-A flies significantly reduced cell viability after 24, 48 and 72 hours of incubation when compared to extracts from TTRwt or control flies. The toxicity of the extracts from TTR-A/TTR-A and TTR-A/+ flies increased over time with a maximum after 48h of incubation, where cell viability was reduced to 13%±5.2 viable cells. In contrast, extracts prepared from 21 days old TTRwt/TTRwt, TTR-A/+ and TTR-A/TTR-A flies did not show any significant toxic effect.

Analysis of extracts by Western blot technique revealed distinct differences in solubility of TTR isolated from flies of different age (Fig. 5B). TTR from young-fly extracts was more soluble than that from older flies, which aggregated and migrated poorly into the gel.

## Discussion

Protein misfolding and aggregation, with formation of amyloidogenic aggregates and amyloid filaments have become of increasing interest due to their involvement in many debilitating disorders such as Alzheimer's disease, Parkinson's disease and TTR-associated amyloidoses (reviewed by [24], [25], [26]). To investigate the link between amyloidogenesis and tissue targeting of protein aggregates *in vivo*, we established a *Drosophila* model for TTR-associated amyloidosis [21]. Here we analyze the pattern of transgenic expression of normal (TTRwt) and mutated (TTR-A) human TTR and pay particular attention to the ultrastructure of relevant tissues. In flies homozygous for the mutated gene we discovered a correlation between the formation of heavy aggregates of nanospheres inside large, membrane-bound cytoplasmic aggregates in adipose tissue and their reduced toxicity.

**Figure 6:**
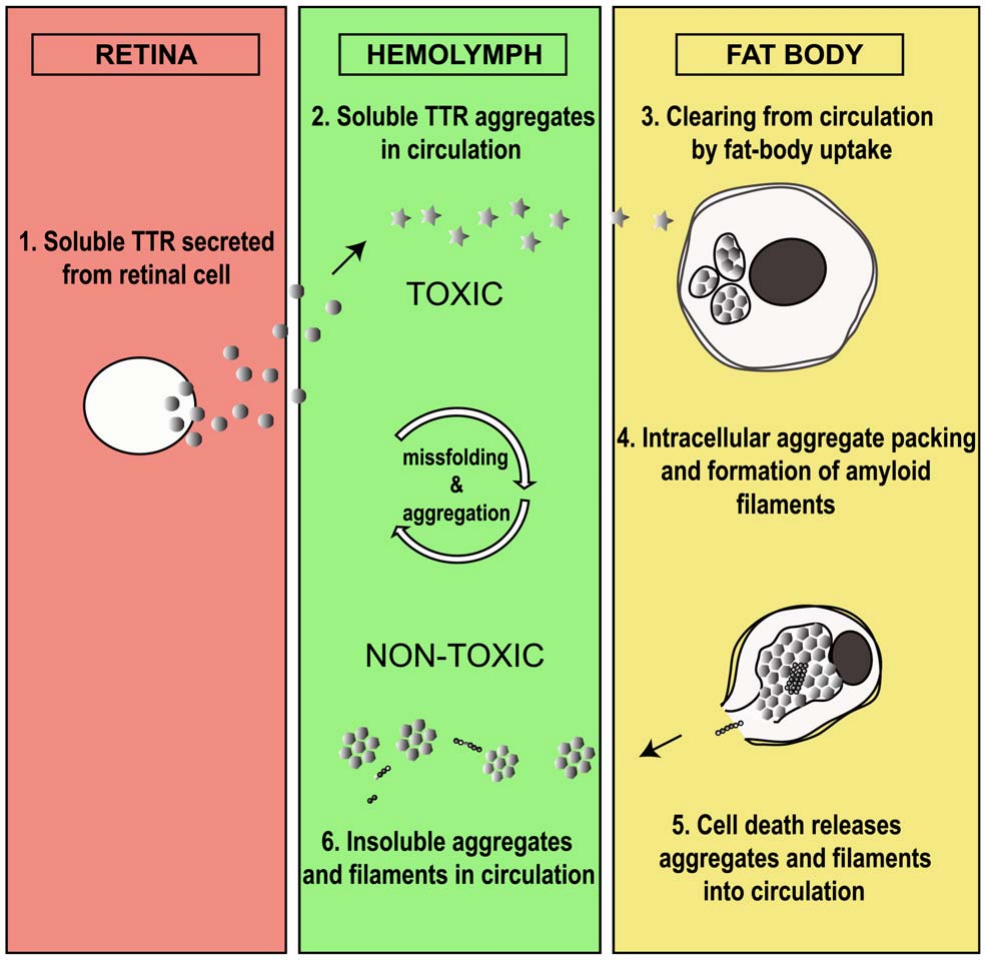
Proposed scenario for the fate of transgenically expressed TTR in the fly. Soluble TTR is expressed in the retina and secreted to the hemolymph where it undergoes misfolding and initial aggregation/oligomerization. Fat body cells take up TTR conformers from the circulation and pack them tightly into aggregates of a few microns size consisting of nanospherules and nanofilaments. This leads to cell death of fat body cells and hypotrophy of the tissue, releasing TTR aggregates and filaments back to the hemolymph. Aggregates formed in the hemolymph at early stages represent the neurotoxic fraction of TTR, whereas aggregates and filaments present in hemolymph at late stages do not exhibit cell toxicity. Thus, the fat body neutralizes toxicity of TTR conformers by uptake from hemolymph and assists their maturation to the detriment of its integrity.

After combining the data from this study with what was previously known, we suggest the following scheme for the fate of transgenically expressed TTR in the fly (Fig. 6). Under the control of the glass promoter (*GMR-Gal4*) the TTR protein is expressed in the retina, from where it is secreted into the hemolymph [21]. Since the retina is the principal source of TTR in our model, retinal degeneration might be relevant for the explanation of the temporal development of the phenotype. In 3 old flies we found no clear signs of retinal degeneration among those expressing either two copies of TTRwt or one copy of TTR-A, and only scarce degenerative features in flies carrying two copies of TTR-A. At 14 days of age, instead, all TTR flies showed signs of retinal degeneration, varying from a mild phenotype in TTRwt flies to strong degeneration in TTR-A flies, especially in those carrying two TTR-A copies.

However, as a note of caution, it has been reported that the expression of GAL4, which is intrinsic to our model, has a dosage and temperature dependent deleterious effect on the expressing cells [27], [28]. To minimize this effect we raised the flies for our experiments at 21–22°C and noted that the gmr-gal4 flies did not develop detectable histological disruption of the retina even at two weeks of age (Figure S5), when the *GMR-Gal4* driver is still active [29].

TTR synthesized in the retina can reach the circulation by two main routes: through secretion directly into the surrounding hemolymph and through disruption of TTR containing cells during retinal degeneration. Our Western blots indicated that part of the soluble TTR reaching the hemolymph is subsequently transformed into aggregates, probably as oligomers (Figure S7). We believe that misfolding and aggregation in the hemolymph result in the formation of the TTR-A species that are responsible for the wing-dragged phenotype and the cytotoxic effect on neuronal cells *in vitro*.

Localization of TTR with or without FLAG as well as the finding of aggregates of nanospherules and nanofilaments in retinal, glial and fat body cells of old TTRwt/TTRwt, TTR-A/+ and TTR-A/TTR-A flies, but not wild-type flies, occurs in tissues that do not express the transgene during adult life (Figure S1). The subretinal glia is in direct contact with the retinal cells [22], and brain glia are known to actively take up cellular debris from surrounding tissue by phagocytosis [30]. It is thus possible that the TTR that is not released directly into the circulation through the disruption of photoreceptors is instead translocated from photoreceptors to subretinal glia through phagocytosis of retinal cell debris.

We also found a punctuated pattern of strong anti-TTR fluorescence in fat body of the head and thorax. This tissue has not been reported to phagocytose cell debris but is specialized for the uptake of proteins from the circulation in both physiological and pathological situations [31]. Therefore, we think that the fly's adipose tissue has the ability to take up TTR from the hemolymph (Fig. 1, 4 and 6). The ultrastructure of TTR-positive tissues was different in head and thorax. In the head, we found aggregates of nanospherules and filaments in brain glia, but not in the fat body. The aggregates observed in glia were not enclosed by a membrane and consisted of long filaments and nanospherules. The aggregates in thoracic fat body, on the other hand, were enclosed by a membrane and contained few and short filaments, and nanospherules tightly packed in a hexagonal array. These structures were exclusively found in old flies carrying two TTR-A copies. The difference between fat body of head and thorax is consistent with the current understanding of the insect fat body as a tissue with morphological and functional regionalization [32], [33].

A hexagonal array represents the most efficient way to pack spherical bodies and in the 2 weeks old TTR-A/TTR-A flies these arrays have a quasi-crystalline structure. We consider that proliferation of these aggregates, and growth beyond the range of 5 or 6 microns will probably challenge the physical integrity of the cell. In some ultrathin sections, we found profiles indicative of disrupted cells. We do not consider them as preparation artifacts, rather the correlation with the hypotrophy of the adipose tissue, the finding of apoptotic nuclei, and the abundance of empty spaces with the size and shape of a single cell, lead us to propose that the large aggregates cause cell damage and the consequent release of aggregates into circulation (“Bursting cell”, Fig. 4G).

An interesting question is whether the TTR species released through cell death are as toxic as the soluble forms present in the circulation before uptake by the fat body. Spherules in the same size range of those reported here have been described to be formed *in vitro* by different proteins, including TTR [34], and the amyloid beta peptide (Aβ) in form of the amyloid-derived diffusible ligands (ADDLs). The latter have been postulated to be the main cause of pathology in the brain of Alzheimer's disease patients [19] and are neurotoxic *in vitro* [35]. It has been found that nanospherules of Aβ (termed amylospheroids) with a diameter between 10 and 15 nanometers were much more toxic than those smaller than 10 nanometers [15], [36]. Thus, the size of oligomers/aggregates might be important for the toxic properties of these assemblies. In the case of TTR, small toxic oligomers have been reported to be formed *in vitro*, which correspond to size not bigger than hexamers (<100 kDa) [14]. However, such cytotoxic species have not been found *in vivo*, although indirect evidence has been presented [16]. Our TTR flies present a potential tool for studies of *in vivo* TTR-derived amyloid formation and its further tissue targeting. Moreover, this model presents a potential in characterizing the neurotoxic fraction of TTR present in the hemolymph.

Amyloid fibrils are currently described as unbranched filaments between 7 and 12 nanometers in diameter. TTR fibrils found in biopsies from FAP patients [37] and TTR fibrils generated *in vitro* are calculated to measure between 8 and 13 nm in diameter [12], [37]. These definitions and dimensions correspond well with the filaments that we found in fat body and brain glia of TTR flies.

The levels of TTR-A are higher in TTR-A/TTR-A than TTR-A/+ flies [21]. This correlates with the severity of damage to the retina and the fat body of TTR-A/TTR-A flies but contrasts with the penetrance of the wing-dragged phenotype, which was more frequent in TTRA/+ flies [21]. Further studies on the aggregation states and their toxicity in different developmental stages and in different tissues are needed to explain the paradoxical finding of an early wing phenotype without ultrastructural pathology in the thoracic tissues. TTR is found very frequently around peripheral nerves in FAP patients and in leptomeninges in TTR-associated CNS amyloidosis. Although the initial definition of amyloid was extracellular deposition of proteinaceous fibrillar material, the use of the term has changed as more evidence exists on the presence of intracellular aggregates of i.e. Aβ in AD [38], [39], [40] and islet amyloid polypeptide-derived amyloid in type II diabetes [41]. Therefore, our *Drosophila* model may shed light on the role of early formation of intracellular TTR amyloid in relevant tissues involved in pathology of ATTR that has been overlooked due to late clinical diagnosis.

## Materials and Methods

### Transgenic contructs and *Drosophila* stocks

The w*;P{w^+mC^ = GAL4-ninaE.GMR}12, abbreviated *GMR-Gal4* [42], obtained from the Bloomington Drosophila Stock Center, Indiana University, was the only gal4 driver used in this study. Expressing lines were generated using standard mating schemes as described previously [21]. Wild-type Oregon R and *w^1118^* flies were used as morphology controls for TEM studies.

TTRwt- and TTR-A-FLAG constructs were generated by introducing the FLAG-tag sequence (GATTACAAGGATGACGACGATAAG) between the TTR signal peptide and the respective TTR gene of previously cloned *pUAST-TTR* vectors [21] with the following primers: TTR-EBprimer 5′-CCGGAATTCCGGATGGCTTCTCATCGTCTGCTC-3′, TTR-FLAGupstream 5′-CCCTTATCGTCGTCATCCTTGTAATCAGCCTCAGACACAAATACCAGTCC-3′, TTR-FLAGdownstream 5′-GCTGATTACAAGGATGACGACGATAAGGGCCCTACGGGCACCGGTG-3′ and TTR-BEprimer 5′-GGAAGATCTTCCTCATTCCTTGGGATTGGTGAC-3′.

The resulting PCR fragments were cloned into the *pCR4-TOPO* vector (Invitrogen), sequenced and then EcoRI-BglII fragments were subcloned into *pUAST* vector (sequenced). Transgenic flies were generated using standard methods [43]. All flies were cultured on standard mashed-potato / yeast / agar media at 21–22°C under 12/12-h light–dark cycles.

Flies of following genotypes were used in the study and abbreviated for practical purposes: *w; GMR-Gal4/GMR-Gal4; +/+* (control flies), *w; GMR-Gal4/GMR-Gal4; UAS-TTRwt/UAS-TTRwt* (TTRwt/TTRwt), *w; GMR-Gal4/GMR-Gal4; UAS-TTR-A/+* (TTR-A/+), *w; GMR-Gal4/GMR-Gal4; UAS-TTR-A/UAS-TTR-A* (TTR-A/TTR-A), *w; GMR-Gal4/GMR-Gal4, UAS- ^FLAG^TTR-A/+* (^FLAG^TTR-A/+), *w; GMR-Gal4/GMR-Gal4; UAS- ^FLAG^TTR-A/UAS- ^FLAG^TTR-A* (^FLAG^TTR-A/^FLAG^TTR-A).

### Immunohistochemistry and fluorescent microscopy

Semi-thin cryosections (10µm) of 14 days old fly heads were fixed with 4% (w/v) formaldehyde in phosphate-buffered saline (PBS) pH 7.3 and incubated in 10% (v/v) fetal calf serum blocking buffer. For studies of thoracic fat body (adipose tissue) the flies were dissected in PBS and the adipose tissue attached to the dorsal epidermis and cuticle was fixed and blocked as above. Immunostainings were performed overnight at 4°C with mouse monoclonal anti-FLAG M2 antibody (1∶500; F1804 Sigma-Aldrich Sweden AB) and Rhodamine Red-X-AffiniPure Goat Anti-Mouse IgG secondary antibody (1∶250; Jackson ImmunoResearch Laboratories, Inc., West Grove, PA, USA). All specimens were mounted on slides with VECTASHIELD mounting medium containing DAPI to counter-stain nuclei (Vector Laboratories, Burlingame, CA). Fluorescent images were collected by either Nikon Eclipse 90i microscope with NIS-Elements Advanced Research (v 3.0) imaging software (NIKON Corporation, Tokyo, Japan) or Zeiss META 510 Confocal microscope with LSM Software (Carl Zeiss GmbH Jena, Germany), and further analyzed and assembled in Adobe Photoshop and Illustrator CS2, respectively.

### Transmission electron microscopy

The flies were anesthetized with nitric oxide (Sleeper TAS, INJECT+MATIC, Switzerland), decapitated with a needle and thereafter the head and thorax were dissected with forceps and needles in a droplet of 0.1 M PBS pH 7.3 on a clean microscope slide. The proboscis and occipital cuticle of the head were gently removed to improve fixative penetration. The dorsal 1/3 of the thorax was dissected to study flight muscles, peripheral nerves and thoracic adipose tissue. The tissues were fixed in a freshly prepared ice-cold solution containing 2.5% glutaraldehyde and 4% formaldehyde in 0.1 M PBS pH 7 for three hours. The samples were then rinsed 4×15 min in PBS, post-fixed for 1 hour in an aqueous 2% solution of osmium tetroxide, rinsed in water, dehydrated in a gradual series of ethanol and acetone and embedded in EPON (EPON 812 embedding kit 3132, Tousimis) according to the manufacturer's instructions. The samples were placed in moulds with pure resin for polymerization at 60°C for 48 hours. Semi-thin sections (around 2 µm) were cut with a glass knife, mounted on microscope slides, stained with 0.1% boracic toluidine blue and used to study the histology and for localization of appropriate sites for ultrastructural analysis. Ultrathin sections (50 to 60 nm) were cut with a diamond knife, contrasted with lead citrate and uranyl acetate and observed with a JEOL JEM 1010 operated at 80 kV. Images were taken with a digital camera (Hamamatsu C4742–95); measurements and image processing were done with the softwares AMT Advantage CCD and Adobe Photoshop. Three flies of each genotype and age (3 or 14–17 days old) were processed: Oregon R and *w^1118^* as normal controls, *w; GMR-Gal4/GMR-Gal4; UAS-TTRwt/UAS-TTRwt, w; GMR-Gal4/GMR-Gal4; UAS-TTR-A/TM3SerSb, w; GMR-Gal4/GMR-Gal4; UAS-TTR-A/UAS-TTR-A*.

### Preparation of fly extracts and immunoblot analysis

2 and 21 days old flies were prewashed in PBS and subsequently sterilized in 70% (v/v) ethanol, which was then drained by capillary forces by placing the flies on sterile Kleenex tissue. Groups of 100 flies of each genotype and age were decapitated with a 27-gauge needle and the abdomens were opened with tweezers in 600 µl of sterile MilliQ water supplemented with 200 U/ml penicillin and 200 µg/ml streptomycin (2× PeSt) as well as 2 µg/ml amphotericin B (GIBCO™ Fungizone®, Invitrogen, Carlsbad, CA). In that way the water extracts were enriched in hemolymph and fat body cells content. All samples were collected in sterile 1.5 ml Eppendorf tubes and stored overnight at 4°C until they were centrifuged at 2500×*g* for 5 min. The supernatants were collected and mixed with supplemented cell culture media in a 1∶11 ratio. 10 µl of each type of fly extracts were mixed with 10 µl 2× loading buffer (4% (w/v) SDS, 125 mM Tris pH 7,5; 30% (v/v) glycerol, 0.2% (w/v) bromophenol blue), without reducing agent, vortexed and separated on Novex® 10–20% Tricine pre-cast mini protein gel (Invitrogen) at 120 V in 1× Tricine running buffer. Proteins were transferred electrophoretically onto Hybond-C Extra Nitrocellulose membrane (0.45 µm pore size; Amersham Biosciences, Buckinghamshire, UK) using a semi-dry system (Trans-Blot® SD, Bio-Rad Laboratories, Hercules, CA). Non-specific binding sites were blocked by incubating membranes overnight at 4°C in 20 mM Tris-HCl (pH 7.5, 137 mM NaCl, and 0.1% Tween-20 (TBS-T) with 5% non-fat dried milk (Semper). Membranes were incubated for 2 h at room temperature with polyclonal rabbit anti-TTR (1∶5000; DAKO, Glostrup, Denmark) and then washed 3×5 min in TBS-T and incubated for 1 h with horseradish peroxidase-labeled goat anti-rabbit antibody (1∶10000; Pierce, Rockford, IL, USA). The immunoreaction was detected with SuperSignal West Pico chemiluminescent substrate (Pierce).

### Cell Culture

Human neuroblastoma IMR-32 cells (CCL-127) were obtained as stock passage number 55 from the American Type Culture Collection (Manassas, VA) and cultured, unless otherwise stated, in Eagle's minimum essential medium (EMEM) with GlutaMAX™ I and Earle's salt, supplemented with 10% fetal bovine serum (FBS), 100 U/ml penicillin and 100 µg/ml streptomycin, 1 mM sodium pyruvate and 0.1 mM nonessential amino acids (Gibco cell culture, Invitrogen, Sweden) at 37°C in a humidified atmosphere of 5% (v/v) CO_2_/air.

### Fly-extracts toxicity assays

IMR-32 cells were seeded at a density of 40000 cells/cm^2^ in 96-well plates and grown in a humidified atmosphere of 5% (v/v) CO_2_/air for 24 hours prior to addition of fly extracts in supplemented cell culture media without phenol red or FBS in a 1∶11 ratio corresponding to 1.5 fly content per 100µl cell culture media per well. The media from the cells was removed, and 100 µl of the fly extracts at appropriate dilution were immediately added to the cells in triplicate. At least six wells containing cells and three wells without cells received 100 µl of media without extracts to serve as cell controls and blanks, respectively. Extracts from flies without expression of TTR (*GMR-Gal4; +*) were used as the negative controls in the toxicity assays, since we observed increased cell viability in cultures treated with fly extract (probably associated with the presence of fly hemolymph proteins), compared to cells growing with medium only. After addition of fly extracts (or media), the cells were incubated at 37°C in a 5% CO_2_ atmosphere for another 18, 24, 48 and 72 hours, when cell viability tests were performed using a resazurin reduction test. Resazurin (blue and non-fluorescent) is reduced to resorufin (pink and highly fluorescent) in living cells [44]. Fluorescence measurements were performed for the viability assay using a Tecan Infinity plate reader with excitation at 530 nm and emission at 590 nm. Each toxicity assay was done a minimum of three times.

### Statistical analysis

Statistical analysis was performed using SPSS 12.0.01 for Windows (SPSS Inc., Chicago, IL, USA). Statistical significance was determined by one-factor analysis of variance (ANOVA) followed by Bonferroni's post hoc. The mean difference was considered to be statistically significant at the 95% confidence level.

## Supporting Information

Figure S1 Expression patterns of GMR-Gal4 driver visualized with UAS-GFP-CAAX reporter during development. A–C, F. The expression of membrane-bound GFP was analyzed under a dissecting microscope with a GFP filter. (A) Strong GFP expression was found in the eye disks (ed) and Bolwig's organ (bo) of the larva, exactly as reported for the expression pattern of gmr (Moses and Rubin 1991). (A′) merged picture of (A) and contrast light analysis. In the pupa (B–C), strong GFP was observed in the developing eye and salivary glands (sgl). A weak autofluorescence was detected in the fat body cells of late pupa. B–H (and not F) In the adult fly (D–E and G–H), immunodetection of GFP in dissected tissues using a mouse monoclonal anti-GFP antibody (1∶1000) revealed expression in the retina (phr, photoreceptors in D), epithelial cells of the wing (E) and haltere (G) and in the salivary glands (H). GFP fluorescence was also observed in the crop (F). Abbreviations: bo, Bolwig's organ; cr, crop; ec, epithelial cells; eo, esophagus; h, haltere; phr, photoreceptors (in the retina); sgl, salivary gland.
(1.98 MB TIF)

Figure S2 Expression of FLAG-tagged TTRwt and TTR-A in fly head extracts. TTR immunodetection was performed with TTR specific polyclonal antibody (DAKO). Extracts of 1,3 heads were loaded per lane of the following genotypes: Lane 1: wild-type flies w1118; lanes 2–4 FLAG-TTR-A/FLAG-TTR-A; lanes 5–6: FLAG-TTRwt/FLAG-TTRwt; lane 7: TTRwt/TTRwt; and lane 8: recombinant TTR (rec.TTR); m, monomer; d, dimer.
(2.04 MB TIF)

Figure S3 Confocal analysis of FLAGTTR-A aggregate formed in thoracic fat body. The aggregate was immunostained with FLAG specific monoclonal antibody. Z-depth of confocal sections (1 µm each) are indicated to the top left of each figure. The TTR-A aggregate measured 8.09 µm as indicated in the zoomed figure in the bottom panel to the right.
(2.99 MB TIF)

Figure S4 Localization of TTR aggregates in transgenic flies. Immunodetection of TTR (in green) with nuclear counterstaining (blue) on paraffin sections is shown. Horizontal head sections of w; GMR-Gal4/+; UAS-TTR-A/+ (A), and w; GMR-Gal4/+; +/+ control flies (B) and fragment of retina with surrounding head fat body cells of w; GMR-Gal4/+; UAS-TTR-A/+ (C). Thoracic fat body of w; GMR-Gal4/+; UAS-TTR-A/+ (D), and indirect flight muscle with surrounding fat body cells (inset in D) of w; GMR-Gal4/ GMR-Gal4; UAS-TTR-A/UAS-TTR-A (E). Retina (Re), fat body (FB) indirect flight muscle (IFM). Aggregates of TTR-A are found in the retina and the thoracic fat body. Arrowheads indicate TTR-positive aggregates. Scale bars, 100 µm in A–B, 20 µm in C–E.
(5.19 MB TIF)

Figure S5 Histology of the head in control (w1118 and GMR-Gal4/GMR-Gal4) and transgenic (TTRwt, TTR-A/+ and TTR-A/TTR-A) flies. At least three heads of each genotype were analyzed in flies of “young” (3 days old) or “old” (14 days old) samples. Representative sections for young and old flies are shown along the top and bottom rows for each genotype, respectively. The retina (Re) showed severe signs of massive degeneration in old TTR-A/+ and TTR-A/TTR-A flies and milder disruption in TTRwt/TTRwt flies.
(1.44 MB TIF)

Figure S6 Electron microscopy of nerves and muscles in TTR-expressing flies. Neither nanofilaments, nanospherules nor other abnormal ultrastructural features were observed in any of the TTR-expressing flies regardless of age. In wild type (A, C), TTRwt (not shown) and TTR-A/TTR-A flies (B, D), we found the normal arrangement of axons (Ax), surrounded by concentric layers of glial cells (Gl) forming the septate junctions that function as blood-nerve-barrier (arrows). In transverse sections of indirect flight muscles (dorsal longitudinal muscles are shown here) the arrangement of myofibrils (Myo), mitochondria (Mi), the dyads formed by the sarcoplasmic system and T-tubules (arrows) and the distribution of thick and thin filaments observed at higher magnification (not shown) appeared normal in TTR-A/TTR-A flies (D) compared with wild type flies. The scale bar shows 500 nm in A–D.
(4.83 MB TIF)

Figure S7 Analysis of oligomeric fraction of TTR. Fly extracts (hemolymph enriched in thoracic fat body content) were separated on 12% Criterion gel under non-reducing conditions from 2 days old (left panel) and 3 weeks old (right panel) flies. TTR immunodetection was performed with TTR specific polyclonal antibody (DAKO). TTR-mers are expected to migrate at following molecular sizes: monomers = 16 kDa, dimers = 28 kDa, trimers = 35 kDa, tetramers = 56 kDa. The extracts were prepared from flies of the following genotypes: Lane 0: control flies, lane 1: TTRwt/TTRwt, lane 2: TTR-A/+, and lane 3: TTR-A/TTR-A. Only TTRwt migrates as monomers, dimers and tetramers. TTR-A shows different from TTRwt migration pattern with a distinct atypical band between 17 and 28 kDa.
(2.49 MB TIF)
