# Supplementary figures and images for: Uptake of Aggregating Transthyretin by Fat Body in a Drosophila Model for TTR-Associated Amyloidosis
Source: PLoS One. 2010 Dec 16;5(12):e14343. doi: 10.1371/journal.pone.0014343 (PMC3002944; doi:10.1371/journal.pone.0014343)

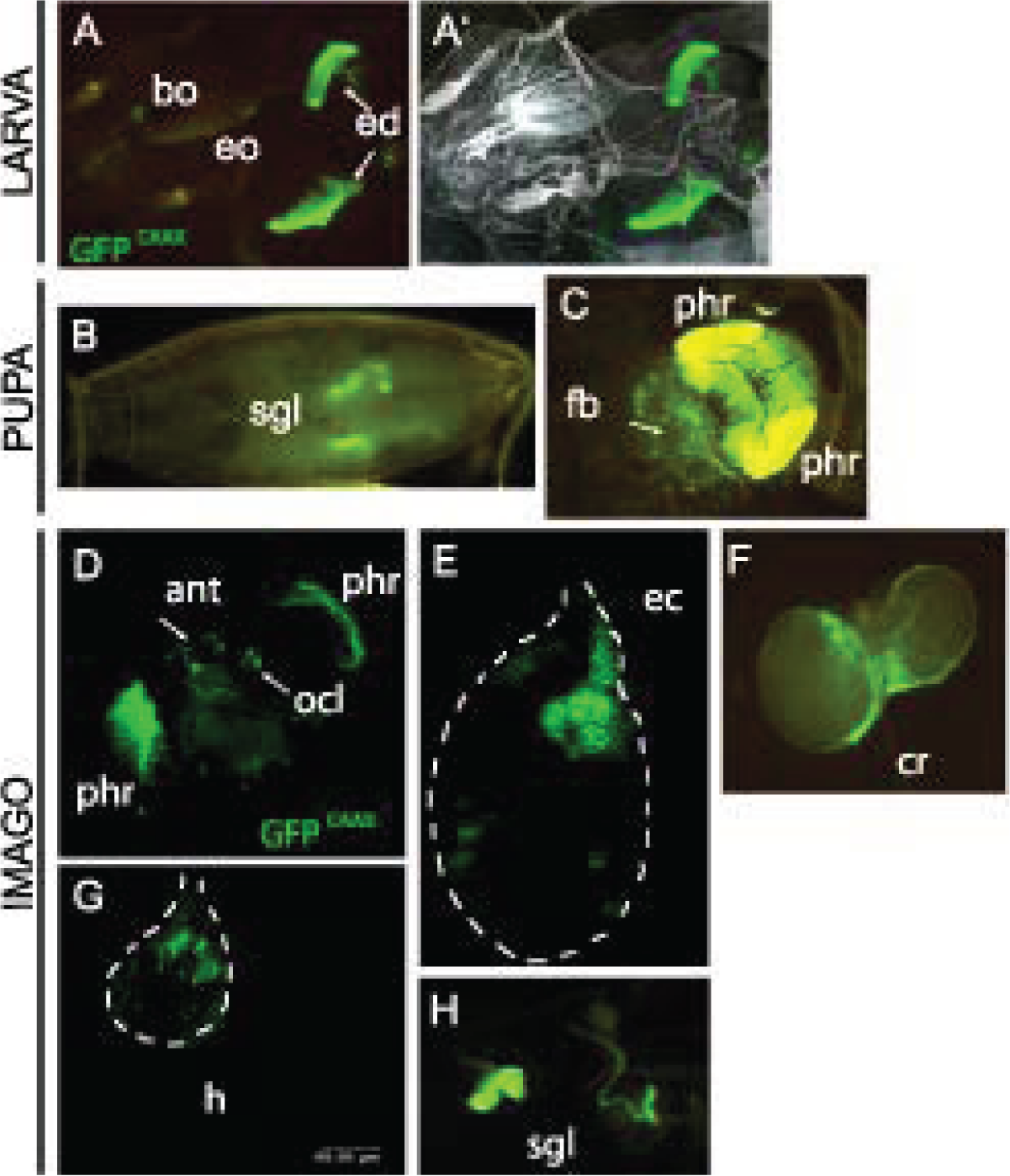

Supplement: Figure S1 — Expression patterns of GMR-Gal4 driver visualized with UAS-GFP-CAAX reporter during development. A–C, F. The expression of membrane-bound GFP was analyzed under a dissecting microscope with a GFP filter. (A) Strong GFP expression was found in the eye disks (ed) and Bolwig's organ (bo) of the larva, exactly as reported for the expression pattern of gmr (Moses and Rubin 1991). (A′) merged picture of (A) and contrast light analysis. In the pupa (B–C), strong GFP was observed in the developing eye and salivary glands (sgl). A weak autofluorescence was detected in the fat body cells of late pupa. B–H (and not F) In the adult fly (D–E and G–H), immunodetection of GFP in dissected tissues using a mouse monoclonal anti-GFP antibody (1∶1000) revealed expression in the retina (phr, photoreceptors in D), epithelial cells of the wing (E) and haltere (G) and in the salivary glands (H). GFP fluorescence was also observed in the crop (F). Abbreviations: bo, Bolwig's organ; cr, crop; ec, epithelial cells; eo, esophagus; h, haltere; phr, photoreceptors (in the retina); sgl, salivary gland. (1.98 MB TIF) [file pone.0014343.s001.tif]

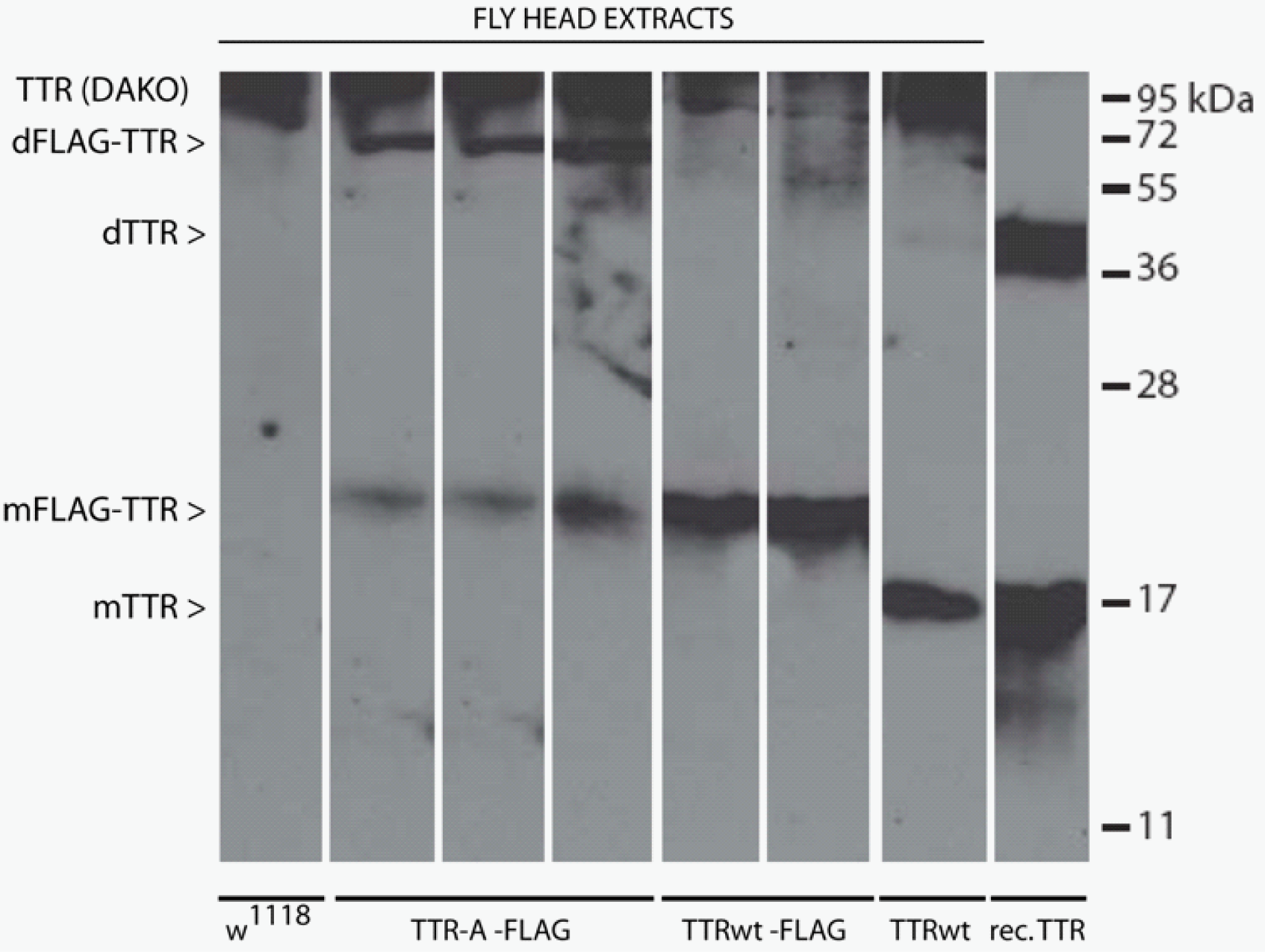

Supplement: Figure S2 — Expression of FLAG-tagged TTRwt and TTR-A in fly head extracts. TTR immunodetection was performed with TTR specific polyclonal antibody (DAKO). Extracts of 1,3 heads were loaded per lane of the following genotypes: Lane 1: wild-type flies w1118; lanes 2–4 FLAG-TTR-A/FLAG-TTR-A; lanes 5–6: FLAG-TTRwt/FLAG-TTRwt; lane 7: TTRwt/TTRwt; and lane 8: recombinant TTR (rec.TTR); m, monomer; d, dimer. (2.04 MB TIF) [file pone.0014343.s002.tif]

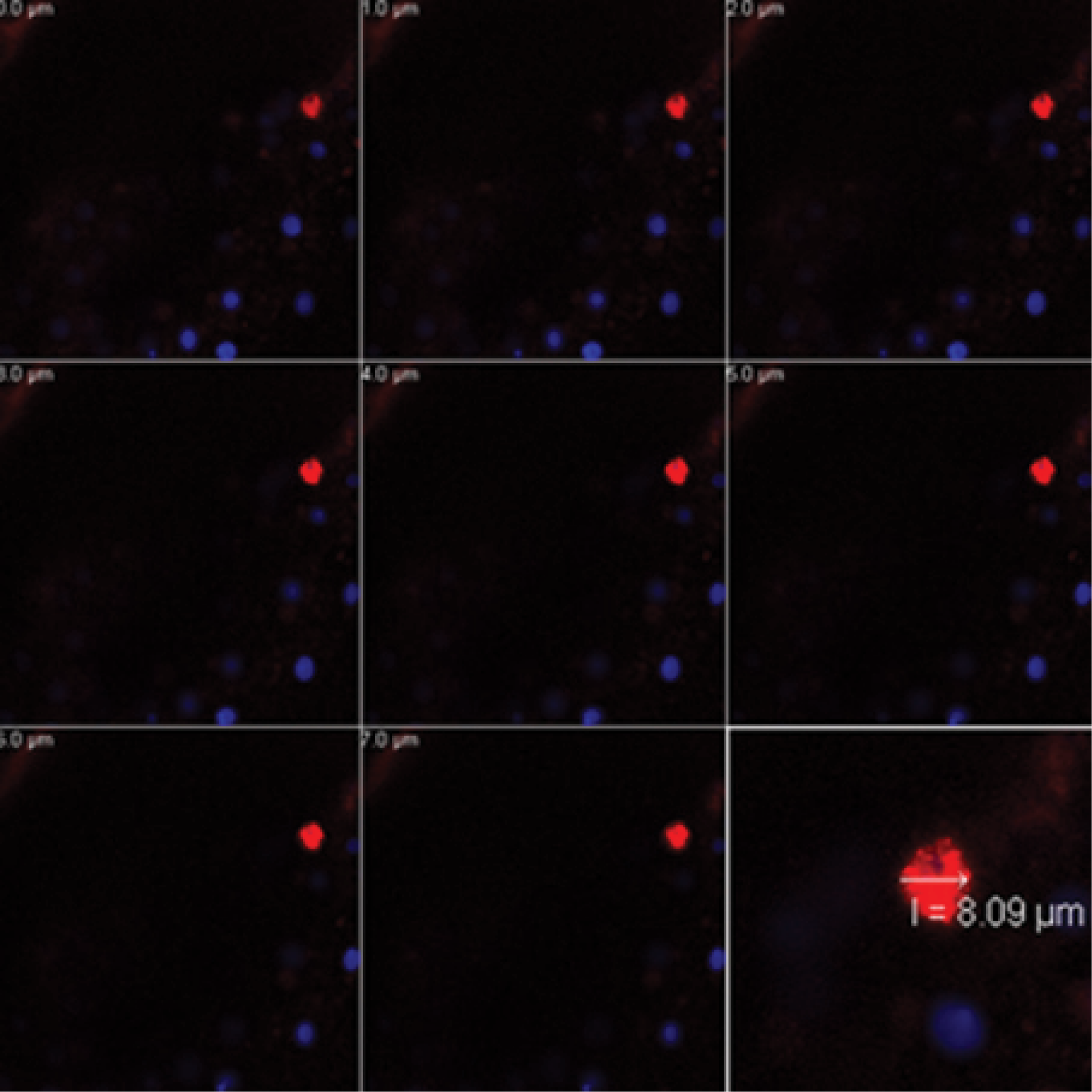

Supplement: Figure S3 — Confocal analysis of FLAGTTR-A aggregate formed in thoracic fat body. The aggregate was immunostained with FLAG specific monoclonal antibody. Z-depth of confocal sections (1 µm each) are indicated to the top left of each figure. The TTR-A aggregate measured 8.09 µm as indicated in the zoomed figure in the bottom panel to the right. (2.99 MB TIF) [file pone.0014343.s003.tif]

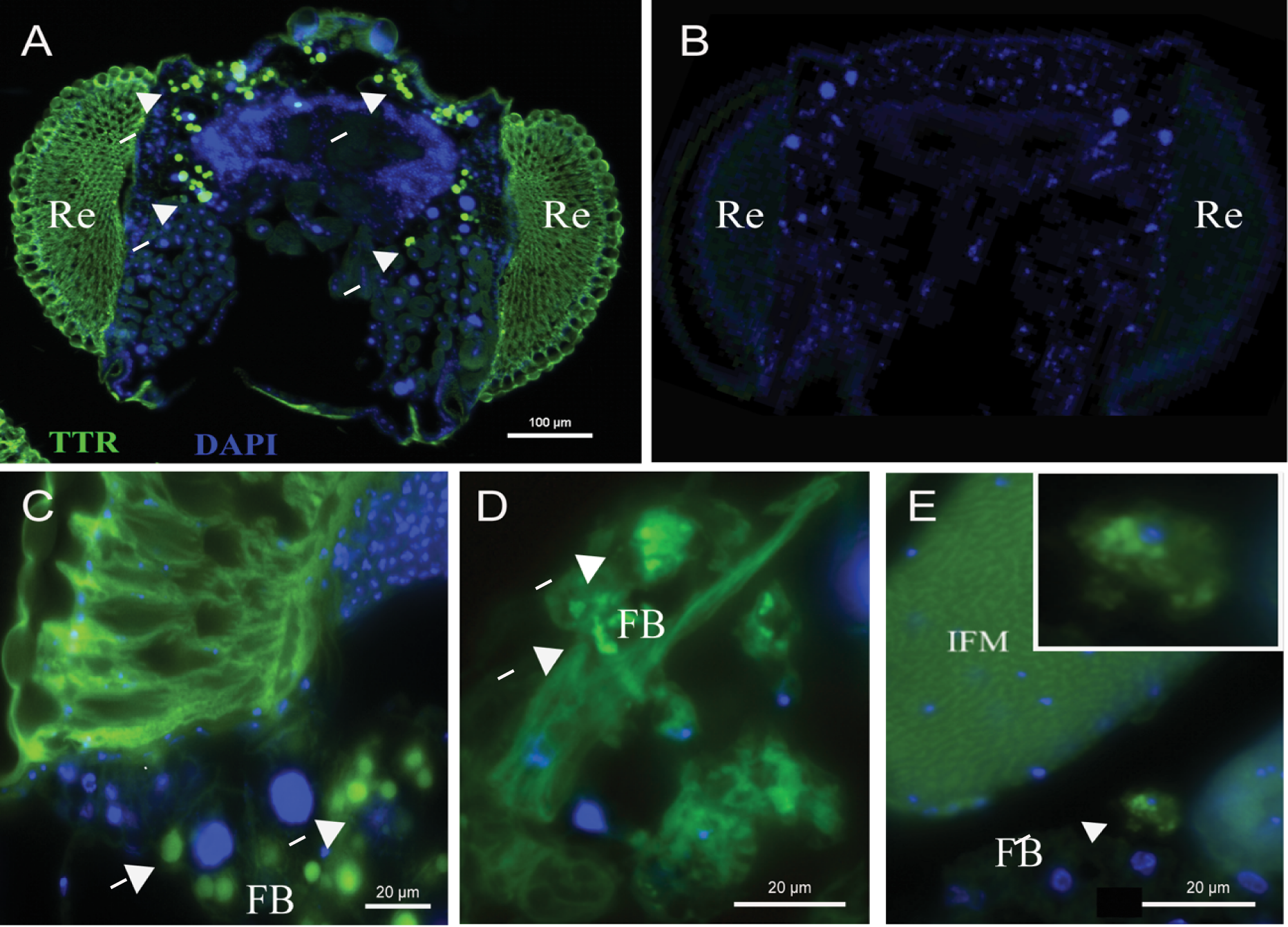

Supplement: Figure S4 — Localization of TTR aggregates in transgenic flies. Immunodetection of TTR (in green) with nuclear counterstaining (blue) on paraffin sections is shown. Horizontal head sections of w; GMR-Gal4/+; UAS-TTR-A/+ (A), and w; GMR-Gal4/+; +/+ control flies (B) and fragment of retina with surrounding head fat body cells of w; GMR-Gal4/+; UAS-TTR-A/+ (C). Thoracic fat body of w; GMR-Gal4/+; UAS-TTR-A/+ (D), and indirect flight muscle with surrounding fat body cells (inset in D) of w; GMR-Gal4/ GMR-Gal4; UAS-TTR-A/UAS-TTR-A (E). Retina (Re), fat body (FB) indirect flight muscle (IFM). Aggregates of TTR-A are found in the retina and the thoracic fat body. Arrowheads indicate TTR-positive aggregates. Scale bars, 100 µm in A–B, 20 µm in C–E. (5.19 MB TIF) [file pone.0014343.s004.tif]

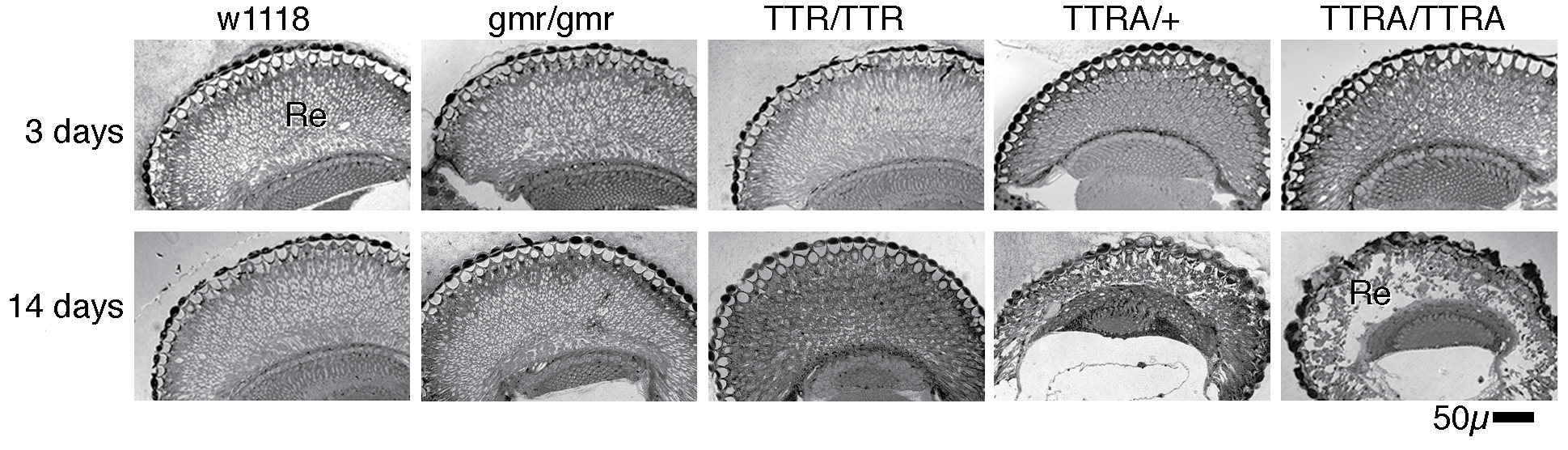

Supplement: Figure S5 — Histology of the head in control (w1118 and GMR-Gal4/GMR-Gal4) and transgenic (TTRwt, TTR-A/+ and TTR-A/TTR-A) flies. At least three heads of each genotype were analyzed in flies of “young” (3 days old) or “old” (14 days old) samples. Representative sections for young and old flies are shown along the top and bottom rows for each genotype, respectively. The retina (Re) showed severe signs of massive degeneration in old TTR-A/+ and TTR-A/TTR-A flies and milder disruption in TTRwt/TTRwt flies. (1.44 MB TIF) [file pone.0014343.s005.tif]

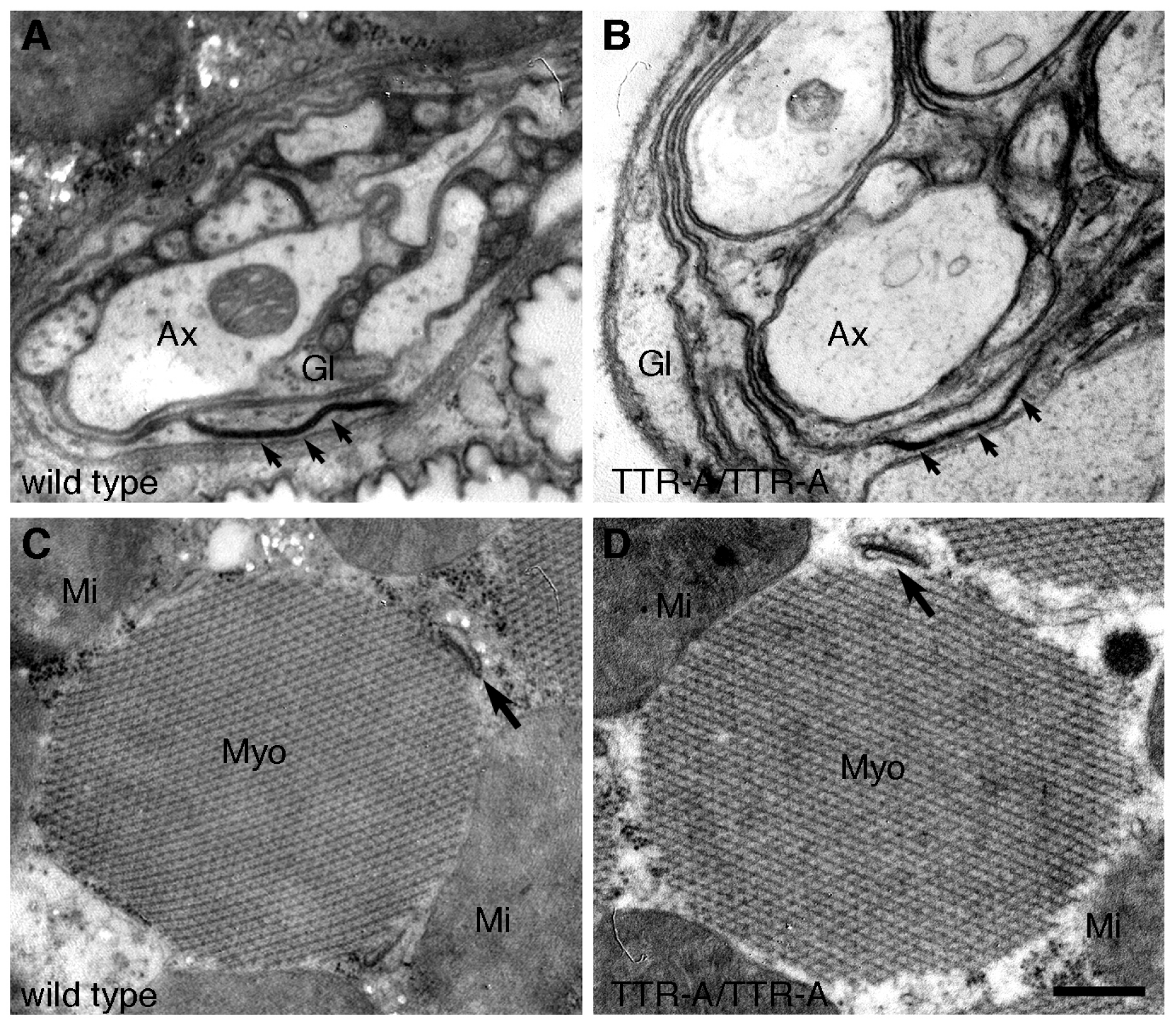

Supplement: Figure S6 — Electron microscopy of nerves and muscles in TTR-expressing flies. Neither nanofilaments, nanospherules nor other abnormal ultrastructural features were observed in any of the TTR-expressing flies regardless of age. In wild type (A, C), TTRwt (not shown) and TTR-A/TTR-A flies (B, D), we found the normal arrangement of axons (Ax), surrounded by concentric layers of glial cells (Gl) forming the septate junctions that function as blood-nerve-barrier (arrows). In transverse sections of indirect flight muscles (dorsal longitudinal muscles are shown here) the arrangement of myofibrils (Myo), mitochondria (Mi), the dyads formed by the sarcoplasmic system and T-tubules (arrows) and the distribution of thick and thin filaments observed at higher magnification (not shown) appeared normal in TTR-A/TTR-A flies (D) compared with wild type flies. The scale bar shows 500 nm in A–D. (4.83 MB TIF) [file pone.0014343.s006.tif]

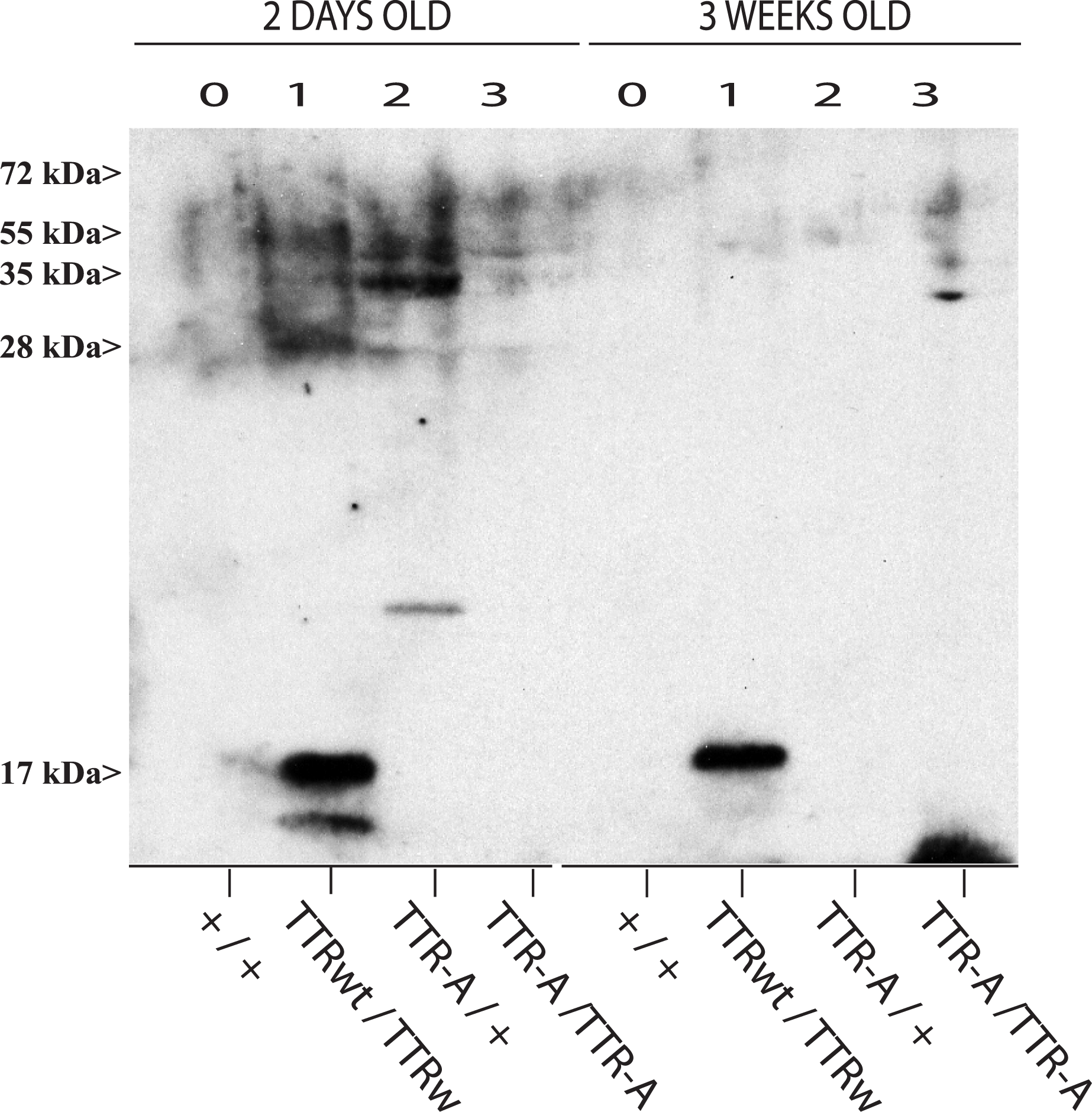

Supplement: Figure S7 — Analysis of oligomeric fraction of TTR. Fly extracts (hemolymph enriched in thoracic fat body content) were separated on 12% Criterion gel under non-reducing conditions from 2 days old (left panel) and 3 weeks old (right panel) flies. TTR immunodetection was performed with TTR specific polyclonal antibody (DAKO). TTR-mers are expected to migrate at following molecular sizes: monomers = 16 kDa, dimers = 28 kDa, trimers = 35 kDa, tetramers = 56 kDa. The extracts were prepared from flies of the following genotypes: Lane 0: control flies, lane 1: TTRwt/TTRwt, lane 2: TTR-A/+, and lane 3: TTR-A/TTR-A. Only TTRwt migrates as monomers, dimers and tetramers. TTR-A shows different from TTRwt migration pattern with a distinct atypical band between 17 and 28 kDa. (2.49 MB TIF) [file pone.0014343.s007.tif]
